# Comparison of Multiplex Real-Time PCR and PCR-Reverse Blot Hybridization Assays for the Direct and Rapid Detection of Porcine Circovirus Type 2 Genotypes

**DOI:** 10.3389/fvets.2020.00200

**Published:** 2020-04-30

**Authors:** Hye-young Wang, Joong Ki Song, Seongho Shin, Hyunil Kim

**Affiliations:** ^1^Optipharm, Inc., Cheongju-Si, South Korea; ^2^Optipharm Animal Disease Diagnostic Center, Cheongju-Si, South Korea

**Keywords:** porcine circovirus type 2, multiplex real-time PCR, PCR-REBA, ORF2, diagnosis

## Abstract

Porcine circovirus type 2 (PCV2), the causative agent of porcine circovirus-associated diseases (PCVAD), poses a serious economic threat for the swine industry. Currently, PCV2 is classified into five major genotypes: PCV2a, PCV2b, PCV2c, PCV2d, and PCV2e. The aim of this study is to evaluate the performance of two commercially available methods, multiplex real-time PCR assay and PCR-reverse blot hybridization assay (REBA), for the rapid detection of PCV2 and direct identification of PCV2 genotypes from clinical samples as well as to compare the results with that of sequence analysis. Molecular diagnostic methods were used to evaluate a total of 180 samples, including tissues and blood samples from pigs that were suspected of PCVAD infection. The results of this study showed that the detection rate for positive PCV2 was 48.3% (*n* = 87) in both multiplex real-time PCR and PCR-REBA methods. Using sequence analysis, which is the gold standard, and multiplex real time PCR assay, the sensitivity, specificity, positive predictive value, and negative predictive value of PCV2 genotyping were found to be 97.1% (*n* = 67, 95% CI 0.894–0.998, *p* < 0.001), 100% (*n* = 93, 95% CI 0.966–1.000, *p* < 0.001), 100% (95% CI 0.953–1.000, *p* < 0.001), 97.9% (95% CI 0.921–0.998, *p* < 0.001), respectively. The results of PCR-REBA were found to be consistent with those of sequence analysis for all the samples and showed good agreement (κ = 1). The most prevalent genotypes detected in this study were PCV2d (*n* = 53, 60.9%), followed by PCV2a (*n* = 17, 19.5%), PCV2b (*n* = 14, 16.1%), and PCV2a/b co-infection (*n* = 3, 3.5%). Both the methods required ~3 h for completion. Therefore, we conclude that two molecular methods are rapid and reliable for the characterization of the causative pathogen with PCV2 genotypes.

## Introduction

Different from the non-pathogenic porcine circovirus (PCV) type 1 strain (1), PCV type 2 is considered to be an important emerging pathogen that causes porcine circovirus associated diseases (PCVAD) including postweaning multisystemic wasting syndrome (PMWS), porcine dermatitis nephropathy syndrome (PDNS), porcine respiratory disease complex (PRDC), enteritis, reproductive failure (2, 3), and one of the most economically important swine diseases worldwide (4). Whenever there are outbreaks of respiratory clinical signs, wasting, and granulomatous inflammation of lymphoid tissues in pigs, PMWS is clinically suspected (5). PCV2-associated systemic infection is clinically characterized by wasting, dyspnea, and lymphadenopathy, and in some cases, might be associated with diarrhea, pallor, and jaundice (6). PCV2, belonging to the genus *Circovirus* of the family *Circoviridae*, is a small non-enveloped virus with a circular single-stranded DNA genome (7). The PCV2 genome is ~1.7 kb nucleotide long and encodes for two open reading frames (ORFs) (8, 9). In the viral genome, ORF1 codes for the replicase (Rep) protein and ORF2 for the capsid (Cap) protein. Rep is a non-structural protein and is responsible for the viral replication, while the structural Cap protein controls the immunogenicity of the virus (10–13). With emerging viral strains, PCV2 has undergone much genetic variation in recent years and has been divided into five genotypes, namely PCV2a-e strains, which are classified based on the diversity level of the ORF2 nucleotide sequences (14, 15). Continuous mutations in the PCV2 genome have made the identification of PCV more difficult, especially by traditional molecular detection methods (16, 17). It has also been demonstrated experimentally that subclinical PCV2 infection might be associated with decreased vaccine efficacy (18). Therefore, PCV2 subclinical infection is not only the most common form of infection in pigs but is also resistant to the effect of vaccines. Hence, rapid and early identification of PCV2 subclinical infection is very important for the effective prophylaxis against PCVAD (19).

Until now, commercial diagnostic tests based on ELISA (9, 12) and PCR (19) have only been developed to confirm the presence or absence of PCV2. Although it has the advantage of being able to detect PCV2 in a short time, it is required expensive antibodies for diagnostic purposes, and the PCV2 genotypes cannot be distinguished simultaneously, so most PCV2 genotypes have been identified separately using PCR-based Restriction Fragment Length Polymorphism (RFLP) (20) or sequence analysis (13, 17, 20–22). In this study, a novel diagnostic assay based on multiplex real-time PCR (Opti PCV2-genotyping; Optipharm, Osong, Republic of Korea) was developed for the rapid and accurate identification of PCV2 as well as to discriminate between the PCV2 a/e, b, and d genotypes. The PCR-based reverse blot hybridization assay (PCR-REBA, REBA PCV2-genotyping; Optipharm) was to detect PCV2 and distinguish between PCV2a, PCV2b, PCV2c, PCV2d, and PCV2e genotypes. In this study, we evaluated the clinical applicability of multiplex real-time PCR and PCR-REBA methods and compared their efficiency to that of the sequence analysis method used for detecting PCV2 and differentiating the different PCV2a-e genotypes directly from the serum and tissue samples of pigs.

## Methods

### Preparation of DNA Samples

To evaluate the diagnostic performance of the multiplex real-time PCR and PCR-REBA methods, a total of 180 samples suspected to be infected with PCVAD including 109 tissues and 71 bloods were provided by the Optipharm Animal Disease Diagnostic Center, which was commissioned from January to December, 2019. DNA was extracted from 200 μL of serum or 20 mg of organ tissue homogenate using a commercial automated system (Miracle-AutoXT Automated Nucleic Acid Extraction System, intronbio, Seongnam, Republic of Korea) according to the manufacturer's recommendations. To avoid cross contamination, all the samples were processed individually and stored at −20°C. The content and purity of the extracted DNA were assayed by measuring absorbance at 260 and 280 nm using an Infinite 200 NanoQuant (Tecan, Switzerland) spectrophotometer.

### Multiplex Real-Time PCR Assay

Detection of PCV2 and identification of genotypes in clinical samples was performed with Opti PCV2-genotyping (Optipharm), a quantitative multiplex real-time PCR-based assay, using the CFX-96 real-time PCR system (Bio-Rad, Hercules, CA, USA) for thermocycling and fluorescence detection. Both the detection and genotype identification of PCV2 can be performed in a single tube using this assay [PCV2 (Cy5), PCV2a/e (FAM), PCV2b/d (CAL Flour Red 610), and PCV2d (HEX)] by incorporating specific TaqMan probes labeled with different fluorophores. Real-time PCR amplification was performed in a total reaction volume of 20 μL containing 10 μL of 2 × Thunderbird probe qPCR mix (Toyobo, Osaka, Japan), 5 μL of a mixture of primer and TaqMan probe that were labeled with different fluorophores, and 5 μL template DNA. The real-time PCR kits consisted of an internal control (IC) DNA, which was used to indicate successful nucleic acid extraction, the quality of the sample and to check for the presence of PCR inhibitors in the reaction. The IC DNA is designed to have minimal sequence similarity with the target gene and also facilitates detection of false negatives. Therefore, it does not directly compete with the amplification of the species-specific target in multiplex real-time PCR. Positive (Plasmid DNA with mixed PCV2a, b, d genotypes) and negative controls consisting of molecular grade (DNAse/RNAse-free) water (Ultra pure water; Welgene, Gyeongsan, Republic of Korea) without template DNA were included in each assay and the assay was performed under the following conditions: 95°C for 3 min followed by 40 cycles of 95°C for 20 s and 55°C for 40 s. Each sample was tested in duplicate by running the PCR cycles twice. The viral load was quantified by determining the cycle threshold (C_T_), and the number of PCR cycles required for the fluorescence to exceed a value significantly higher than the background fluorescence. Positive result was indicated when the C_T_ value was <38.

### PCR-Reverse Blot Hybridization Assay (PCR-REBA)

**Figure 1:**
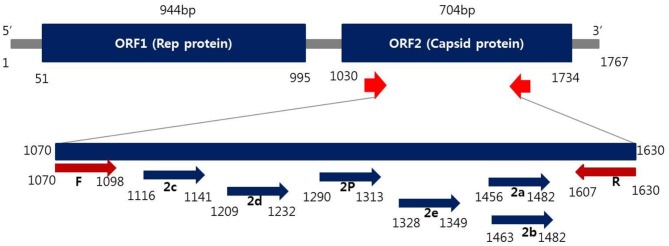
Schematic representation of the ORF2 gene to distinguish PCV2 genotyping from positions of primer and probes used in this study.

Oligonucleotide primers corresponding to both strands of the ORF2 region of PCV2 (Figure 1) were designed by Primer3Plus (http://www.bioinformatics.nl/cgi-bin/primer3plus/primer3plus.cgi). The primers were made as probes corresponding to the complementary strand and were used exclusively thereafter. To validate the efficiency of the selected probes, target DNA samples amplified from the PCV2 strains were applied to the REBA membrane strips and spotted with the selected probes. Two types of DNA samples (PCV2c and PCV2e) were synthesized (Bioneer, Daejeon, Republic of Korea) and amplified with custom PCR primers (PCV2c, F-5′-TAAGTGGGGGGTCTTTAAGA-3′ and R-5′-TCCTCCGCCGCCGCCCCTGG-3′; PCV2e, F-5′-TAAGTGGGGGGTCTTTAA-3′ and R-5′-CTTGGCCATATCCTCCGCC-3′), resulting in amplicons of 630 and 640 bp, respectively. The resultant products were mutagenized after subcloning into the pBHA vector. Two plasmids were extracted from the transformants, and the mutated sequences were confirmed by sequence analysis (CosmoGenetech, Daejeon, Republic of Korea). PCR was performed using a 20 μL reaction mixture (GeNet Bio, Daejeon, Republic of Korea) containing 2 × master mix (10 μL), 2 μL of primer mixture, 5 μL sample DNA, and 3 μL Ultra pure water (Welgene) to make up the final volume. The reactions were run on a Verity thermocycler (Applied Biosystems, CA, USA) under the following conditions: one cycle at 94°C for 5 min, followed by 35 cycles of 94°C for 30 s, annealing 60°C for 30 s, initial extension at 72°C for 30 s, and a final extension of 72°C for 10 min to complete the synthesis of all strands. The amplified target was visualized as a single band corresponding to a length of 620 bp using the ChemiDoc system (Vilber Lourmat, Eberhardzell, Germany).

For REBA PCV2 genotyping, the hybridization and washing processes were performed as follows; each sample was tested in duplicate and all PCR-REBA runs were performed twice. In brief, biotinylated PCR products were denatured at 25°C for 5 min in denaturation solution and then the denatured single-stranded PCR products suspended in hybridization solution were incubated with REBA PCV2-genotyping membrane strips at 55°C with shaking at 90 rpm in a blotting tray for 30 min. The strips were then washed twice with gentle shaking in 1 ml of washing solution for 10 min at 55°C, incubated at 25°C with 1:2,000 diluted streptavidin-alkaline phosphatase (AP) conjugate (Roche Diagnostics, Mannheim, Germany) in conjugate diluent solution (CDS) for 30 min, and finally washed twice with 1 ml CDS at room temperature for 1 min. The colorimetric hybridization signals were visualized by adding a 1:50 dilution of nitro blue tetrazolium chloride/5-bromo-4-chloro-3-indolyl (NBT/BCIP) AP-mediated staining solution (Roche Diagnostics), and then incubated until a color change was detected. Finally, the band pattern was read and interpreted visually.

### Sequence Analysis

To confirm the results of the two molecular diagnostic methods, the PCR amplicons of all the clinical samples were sequenced using an ABI 3730 automated DNA sequencer (Applied Biosystems, Foster City, CA, USA) and the ABI Prism BigDye Terminator (Applied Biosystems) system (CosmoGenetech, Republic of Korea). The primer set used to amplify the target ORF2 gene was 5′-TCTGAATTGTACATACATRGTTAYACGG-3′ (1070F) and 5′- TACCGYTGGAGAAGGAAAAATGG-3′ (1630R), which resulted in a 560-bp PCR product. The obtained sequences were compared with sequences in the National Center for Biotechnology Information (NCBI) GenBank database for species identification.

## Results

### Analytical Sensitivity and Specificity of the Multiplex Real-Time PCR and PCR-REBA Methods

Analytical sensitivity of the two molecular methods for the detection of PCV2 was determined by using 10-fold diluted (1 ng, 100 pg, 10 pg, 1 pg, 100 fg, 10 fg, and 1 fg) samples for obtaining the standard curve for the DNA extracted from PCV2 strains. The detection limit of the multiplex real-time PCR assay for PCV2-P (PCV2 species-specific probe), PCV2a/e-P (PCV2a/e genotype-specific probe), PCV2b/d-P (PCV2b/d genotype-specific probe), PCV2c (PCV2c genotype-specific probe), PCV2d-P (PCV2d genotype-specific probe), and PCV2e-P (PCV2e genotype-specific probe) ranged from 100 to 10 fg DNA per reaction. The C_T_ values for PCV2-P, PCV2a/e, PCV2b/d, PCV2c, PCV2d, and PCV2e for each DNA concentration ranged from 17 to 36.8, 16.5 to 34.9, 16.7 to 35.1, 16.1 to 35, 16.8 to 35.3, and 15.5 to 33.1, respectively (Supplementary Figures 1A–D). The PCR-REBA detection limit for PCV2a, PCV2b, PCV2c, PCV2d, and PCV2e was ~100 fg to 10 fg DNA per reaction (Supplementary Figures 1E–J). In addition, the detection limit for mixed co-infection PCV2 subtypes in multiplex real-time PCR was 1 pg DNA per reaction, and the C_T_ value was found to be 17.97–32.86 (Data not shown). The PCR-REBA detection limit for mixed co-infection PCV2 subtypes detected ~100 fg DNA per reaction (Supplementary Figure 1K).

To determine the specificity of the two molecular assays, primers and probes for detecting PCV2 positive genotypes were used for testing 55 DNA samples, respectively, extracted from specific pathogen-free swine serum samples and used as negative controls. The multiplex real-time PCR and PCR-REBA assay for detecting PCV2 positive genotypes yielded negative results with all strains except PCV2 strains (including mixed co-infection PCV2 subtypes), hence, the cross reactivity was not detected (Supplementary Table 1).

### Detection of PCV2 DNA Using Multiplex Real-Time PCR and PCR-REBA Methods in Clinical Samples

To evaluate the performance of the multiplex real-time PCR and PCR-REBA assay, a total of 180 clinical samples including tissue (*n* = 109, 60.6%) and whole blood (*n* = 71, 39.4%) were analyzed. Of 180 clinical samples, 87 (48.3%) samples were positive for PCV2, and 93 (51.7%) samples were negative as detected by both multiplex real-time PCR and PCR-REBA (Table 1).

**Table 1:** Detection of porcine circovirus 2 DNA in 180 clinical samples suspected of PCVAD infection using the multiplex real-time PCR and PCR-REBA assay.

**Sample**	**Total no. (%) of samples**	**Multiplex real-time PCR**		**PCR-REBA**	
		**Positive (%)**	**Negative (%)**	**Positive (%)**	**Negative (%)**
Tissue	109 (60.6)	54 (49.5)	55 (50.5)	54 (49.5)	55 (50.5)
Blood	71 (39.4)	33 (46.5)	38 (53.5)	33 (46.5)	38 (53.5)
Total	180 (100)	87 (48.3)	93 (51.7)	87 (48.3)	93 (51.7)

### Multiplex Real-Time PCR and PCR-REBA Methods for the Detection of PCV2 Genotyping in Clinical Samples

**Figure 2:**
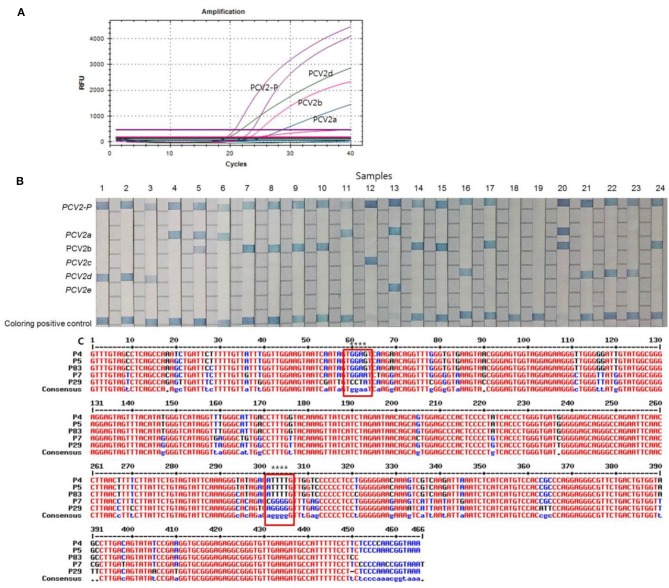
Typical results of the multiplex real-time PCR, PCR-REBA, and sequence analysis with clinical samples. **(A)** Overall results for PCV2-positive, PCV2a, PCV2b, and PCV2d. Fluorescent dyes of specific TaqMan probes for multiplex real-time PCR were used PCV2 (Cy5), PCV2a/e (FAM), PCV2b/d (CAL Flour Red 610), and PCV2d (HEX), respectively. **(B)** Results of PCR-REBA; Lanes 1–3, 16, 21–23: PCV2d; lanes 4, 6, and 11: PCV2a; lanes 5 and 20: PCV2a and PCV2b co-infection; lane 7–10, 14–15, 17, and 24: PCV2b; lane 12: PCV2c; lane 13: PCV2e; lane 18 and 19: negative. PCV2c and PCV2e were used to synthesize the DNA as a control. **(C)** Sequence alignment results of a fragment of the genomic sequence of the clinical samples; P4, PCV2a; P7, PCV2b, P29, PCV2d, P5 and P83 showed that the two samples detected as PCV2a/b co-infection positive by the multiplex real-time PCR and PCR-REBA methods were shown as only PCV2a positive by sequence analysis; The red boxes indicate the position where three genotypes (PCV2a, 2b, and 2d) can be identified.

Of the 87 PCV2 positive samples, 53 (60.9%), 17 (19.5%), 12 (13.8%), and 3 (3.5%) samples showed positive fluorescence signals for PCV2d, PCV2a, PCV2b, and PCV2a/b co-infections, respectively, as evaluated using multiplex real-time PCR assay (Figure 2A) while no PCV2 genotypes were detected in 2 cases (2.3%). All the clinical samples showed positive IC signals and the C_T_ values of the 87 positive and 93 negative samples ranged from 23.62 to 32.7 (mean 24.89, SD ± 1) and 22.47 to 33.4 (mean 25.36, SD ± 0.47), respectively. The C_T_ values of the PCV2d, PCV2a, PCV2b, and PCV2a/b co-infections samples ranged from 15.75 to 35.08 (mean 23.43, SD ± 5.6), 20.97 to 33.85 (mean 26.5, SD ± 4.07), 22.89 to 34.37 (mean 28.81, SD ± 5.78), and 21.2 to 30.73 (SD ± 2.4), respectively. PCR-REBA, which is another method for performing the molecular identification of PCV2 genotypes, was performed with the same clinical samples (Figure 2B). Of the 87 positive samples, the following PCV2 genotypes were identified using PCR-REBA: PCV2d was the most prevalent at 60.9% (*n* = 53), followed by PCV2a (*n* = 17, 19.5%), PCV2b (*n* = 14, 16.1%), and PCV2a/b co-infections (*n* = 3, 3.5%), respectively (Table 2).

**Table 2:** Comparison of multiplex real-time PCR, PCR-REBA, and sequence analysis results for the detection of PCV2 genotypes in 180 clinical samples suspected of PCVAD.

**PCV2 genotyping**	**Molecular methods**							**Sensitivity (%)**	**95% CI**	**Specificity (%)**	**95% CI**
	**PCR-REBA**		**Multiplex real-time PCR**		**Sequence analysis**						
	**Positive (%)**	**Negative (%)**	**Positive (%)**	**Negative (%)**	**Positive (%)**	**N/A (%)**	**Consistent results**				
**PCV2 positive (*****n*** **=** **87)**	**87 (48.3)**	**0 (0)**	**85 (97.7)**	**2 (2.3)**	69 (79.3)*	**18 (20.7)**	**69 (100)**	100	0.954–1.000	100	0.979–1.000
**Single infection (*****n*** **=** **84)**	**84 (96.6)**	**0 (0)**	**82 (96.5)**	**2 (100)**	**67 (97.1)**	**17 (94.4)**	**67 (100)**	100	0.953–1.000	100	0.972–1.000
PCV2d	53 (63.1)	0 (0)	53 (63.1)	0 (0)	42 (79.2)	11 (20.8)	42 (100)	100	0.927–1.000	100	0.884–1.000
PCV2a	17 (20.2)	0 (0)	17 (20.2)	0 (0)	15 (88.2)	2 (11.8)	15 (100)	100	0.784–1.000	100	0.940–1.000
PCV2b	14 (16.7)	0 (0)	12 (14.3)	2 (2.4)	10 (71.4)	4 (28.6)	10 (100)	100	0.787–1.000	100	0.954–1.000
**Multiple infection (*****n*** **=** **3)**	**3 (3.4)**	**0 (0)**	**3 (3.5)**	**0 (0)**	**2 (2.9)**	**1 (5.6)**	**2 (100)**	100	0.425–1.000	100	0.961–1.000
PCV2a and PCV2b	3 (100)	0 (0)	3 (100)	0 (0)	2 (66.7)	1 (33.3)	2 (100)	100	0.369–1.000	100	0.953–1.000
**PCV2 negative (*****n*** **=** **93)**	**0 (0)**	**93 (100)**	**0 (0)**	**93 (100)**	–	–	–	–	–	–	–
**Total (*****n*** **=** **180)**	87 (48.3)	93 (51.7)	85 (47.2)	95 (52.8)	69 (79.3)	18 (20.7)	–	–	–	–	–

*N/A, not applicable*;

* *A total of 18 samples including 11 PCV2d, 2 PCV2a, 4 PCV2b, and 1 PCV2a/b co-infection identified by multiplex real-time PCR and PCR-REBA were excluded because they were not sequenced; 95% CI, 95% confidence interval*.

### Comparison of the Results Between the Two Molecular Assays and Sequence Analysis for Identification of the PCV2 Genotypes in the Clinical Samples

**Figure 3:**
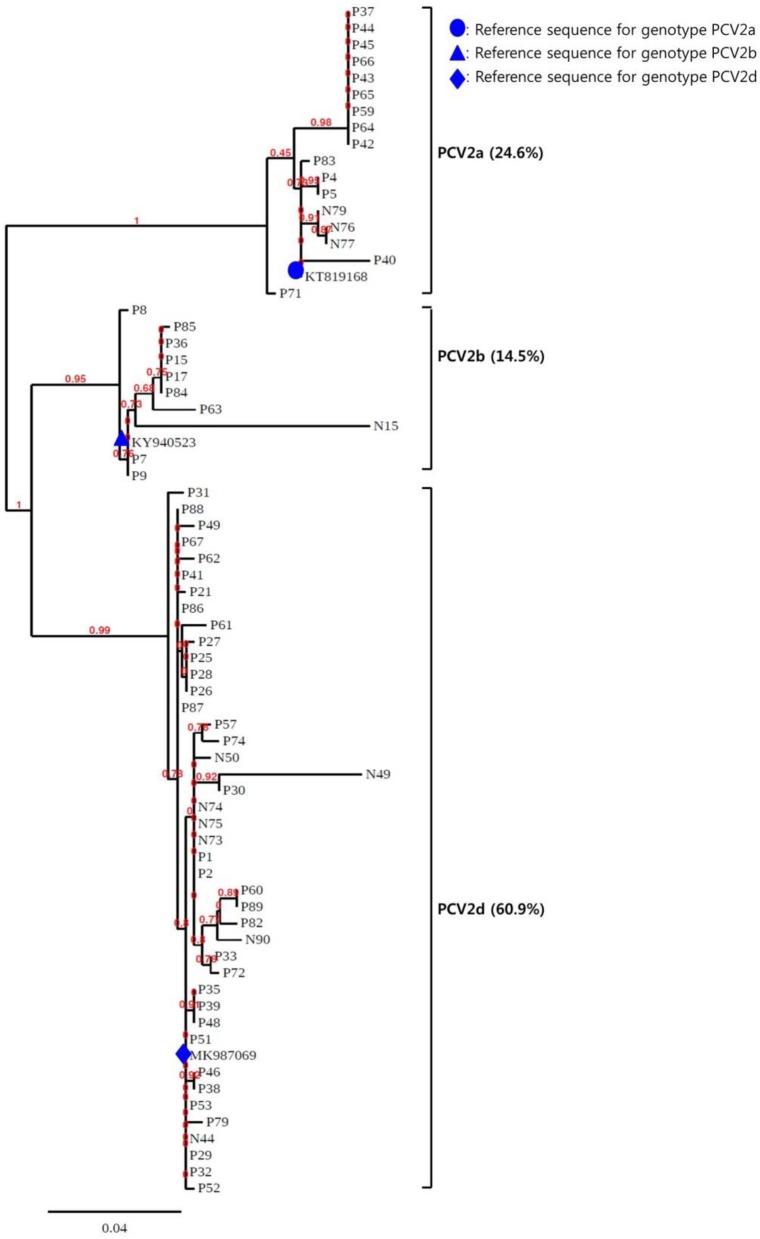
Phylogenetic analysis of the 69 PCV2 isolates. The phylogenetic tree was constructed using Phylogeny.fr software after alignment of the 69 sequenced results. The PCV2genomes were mainly assigned to three genotypes (PCV2a, PCV2b, and PCV2d).

To confirm the results obtained from the multiplex real-time PCR and PCR-REBA assay, sequence analysis was performed using the same clinical samples (Figure 2C). Only 69 (79.3%) of the 87 PCV2 positive samples could be identified for PCV2 genotyping by sequence analysis. Therefore, 18 samples including 11 PCV2d, 2 PCV2a, 4 PCV2b, and 1 PCV2a/b co-infected samples identified by multiplex real-time PCR and PCR-REBA were excluded from the comparative analysis. The results of the multiplex real-time PCR and sequence analysis methods were consistent except for two cases. In these two cases, while PCV2 genotypes were not detected using multiplex real-time PCR, the samples were identified as PCV2b-positive by sequence analysis. The results of the PCR-REBA for PCV2 genotyping of all 69 samples were consistent with the sequencing results (Table 2). The phylogenetic tree was constructed using Phylogeny.fr software (23) after alignment of the 69 sequenced results. Analysis of the phylogenetic tree indicated that the sequences could be divided into three genotypes (PCV2d, PCV2a, and PCV2b), which accounted for 60.9, 24.6, and 14.5%, respectively (Figure 3).

## Discussion

Although the severity of the economic losses caused by PCV2 infection has been mitigated by vaccination, PCVAD is still detected quite often and is an important porcine pathogen. It is important to distinguish between the PCV2 genotypes for the laboratory diagnosis of PCV2 as different PCV2 genotypes have been found in samples from pigs affected with other PCVAD (24). The currently used PCR-based methods for diagnosis, including nucleotide sequencing, PCR, nested PCR, and PCR-RFLP (25–28), are time consuming for general veterinary clinical applications; they can only be used in specialized diagnostic institutes as these methods require specialized equipment and reagents, and need to be inspected by professionals to differentiate the subtype virus. Therefore, there is an increasing requirement for diagnostic techniques that can be complemented with traditional methods in clinical diagnostic laboratories.

In the present study, two molecular assays were developed for detecting PCV2 genotypes. The purpose of the present study was to evaluate the clinical efficacy of the multiplex RT-PCR assay (Opti PCV2-genotyping) and PCR-REBA (REBA PCV2-genotyping) for rapid and accurate detection as well as identification of PCV2 genotypes based on the ORF2 regions. We also compared the results of these two molecular assays with those obtained from conventional methods such as sequence analysis. The characteristics of the multiplex real-time PCR and PCR-REBA used in this study were similar as they were both rapid (turnaround time of 2–3 h), sensitive, specific, and comparatively easy to perform without requiring any specialized laboratory equipment, other than a PCR machine and water bath.

The Opti PCV2-genotyping assay is designed to simultaneously detect PCV2 and to distinguish PCV2a/e, PCV2b, and PCV2d genotypes. Multiplex real-time PCR, a recognized technique, is faster and more effective for the rapid detection of bacterial or viral infection compared to conventional PCR and other detection methods. Multiplex real-time PCR assay is a rapid method with a turnaround time of ~1.5–2 h, which includes 30 min for DNA preparation and 1.5 h for target DNA amplification. The combination of excellent sensitivity and specificity as well as ease of handling enable rapid and simultaneous detection of multiple species, and minimizes the possibility of contamination by eliminating the need for additional post-PCR processing of the samples, which has made this technology appealing for clinical microbiology laboratory applications (29). PCR-REBA is a highly sensitive and specific probe-based method in which multiple oligonucleotide probes are immobilized on nitrocellulose strips, hybridized with biotin-labeled PCR products, and can be used to derive rapid results within 4 h (30). In addition to the time required for target DNA amplification (1.5 h), PCR-REBA is a 3-step process with a hybridization step (30 min), a washing step (20 min), and a chromogenic detection and data interpretation step (40 min). It also requires a fully automated system for the washing, hybridization, and interpretation steps. The PCR-REBA molecular diagnostic assay can be used to isolate all types of PCV2 genotypes as well as detect PCV2 directly from serum or tissue samples. In addition, PCR-REBA has the advantage of the flexibility to add more specific-probes to the membrane strip for increasing the range of PCV2 genotypes detected.

In this study, the concordance rate of the multiplex real-time PCR assay and sequence analysis was 98.8% (95% confidence interval [CI] 0.953–0.999, *p* < 0.001). Using sequence analysis as the gold standard, the sensitivity, specificity, and positive and negative predictive values of the PCV2 genotyping results by multiplex real-time PCR assay were 97.1% (*n* = 67, 95% CI 0.894–0.998, *p* < 0.001), 100% (*n* = 93, 95% CI 0.966–1.000, *p* < 0.001), 100% (95% CI 0.953–1.000, *p* < 0.001), 97.9% (95% CI 0.921–0.998, *p* < 0.001), respectively. The results of PCR-REBA were found to be consistent with those of sequence analysis and showed good agreement (κ = 1).

Studies have shown that the most common PCV2 genotypes detected worldwide are PCV2b (53.1%) and PCV2a (34.4%) in Taiwan (24), PCV2b (87.5%) and PCV2a (12.5%) in Mexico (31), and PCV2d (45.3%) and PCV2b (41.1%) in China (13). In this study, the most prevalent genotypes detected were PCV2d (*n* = 53, 60.9%), followed by PCV2a (*n* = 17, 19.5%), PCV2b (*n* = 14, 16.1 %), and PCV2a/b co-infection (*n* = 3, 3.5%). Co-infection of PCV2a and PCV2b in clinical samples has been suggested to be the primary cause of other PCVAD while dual heterologous infection of PCV2a and PCV2b in gnotobiotic pigs has been shown to induce severe clinical symptoms (24, 32). Therefore, rapid identification of co-infection of PCV2a and PCV2b is crucial. Generally, when identified samples from dually infected pigs were sequenced, only the predominant PCV2 genotype was detected. Our results also showed that only PCV2a could be identified by sequence analysis method in the three samples in which PCV2a/b co-infection was detected using the two molecular diagnostic methods.

There are potential limitations in this study. Firstly, the multiplex real-time PCR assay cannot distinguish between PCV2a type and PCV2e type, and does not include PCV2c type that has not yet been detected in Korea. Therefore, there should be an additional tube to include all of these genotypes. Secondly, although PCR-REBA detect all other genotypes in addition to PCV2, additional steps are required after PCR. Thirdly, the PCV2c and PCV2e genotypes were not detected in this study and further investigations of the samples are required.

## Conclusions

The two recently developed molecular assays are accurate, rapid, and convenient tools for identifying PCV2. These assays can also discriminate between the PCV2 genotypes and directly detect PCV2 from clinical samples in only 2–3 h. Therefore, these two molecular assays can provide essential information that can help expedite therapeutic decisions for early and appropriate vaccinations during the acute phase of PCV2 infection. We believe that these assays can reduce the labor and time for PCV2 diagnosis in industrial animal area.

## Data Availability Statement

The raw data supporting the conclusions of this article will be made available by the authors, without undue reservation, to any qualified researcher.

## Ethics Statement

All samples used in this study were animal diagnostic samples submitted by the clients and there was no animal handling involved.

## Author Contributions

HW performed evaluation of the experiments, analyzed the data, and drafted the manuscript. JS and SS provide clinical samples and clinical information. HK revised the manuscript. All authors have read and approved the final manuscript.

## Conflict of Interest

All authors were employed by Optipharm, Inc during the preparation and execution of the study.

## Supplementary Material

Supplementary Figure 1 Detection limits of the multiplex real-time PCR and PCR-REBA methods evaluated using 10-fold serial diluted samples. Serially diluted PCV2-P, PCV2a/e, PCV2b, and PCV2d DNA samples ranging from 1 ng to 1 fg per reaction were used to determine the detection limit of the multiplex real-time PCR and PCR-REBA methods. In the multiplex real-time PCR assay, the amplification curve of the specific probe **(A)** for detecting PCV2 (*R*^2^ = 0.997), PCV2a/e probe **(B)**, PCV2b probe **(C)**, and PCV2d probe **(D)**, for detecting PCV2 genotypes (*R*^2^ = 0.999) are shown. The overall detection limit of this assay for the PCV2 genotypes ranged from ~100 to 10 fg DNA per reaction. C_T_ was plotted against the input of the quantity of PCV2, 2a/e, 2b, 2c, 2d, and 2e DNA (repeated 40 times). The intensity of fluorescence is shown on the Y-axis (*R*^2^ = reporter signal/passive reference signal). RFU, relative fluorescence unit and *R*^2^, fluorescence units. Serially diluted PCV2-P **(E)**, PCV2a **(F)**, PCV2b **(G)**, PCV2c **(H)**, PCV2d **(I)**, PCV2e **(J)**, and 5 mixed co-infection PCV2 subtypes **(K)** with DNA amounts from 1 ng (lane 1), 100 pg (lane 2), 10 pg (lane 3), 1 pg (lane 4), 100 fg (lane 5), 10 fg (lane 6), and 1 fg (lane 7) were used to determine the detection limit of the PCR-REBA **(E–J)**. N, negative control. PCV2c and PCV2e used synthesized DNA as a control.

Supplementary Table 1 Analytical specificity of the multiplex real-time PCR and PCR-REBA assay for detecting PCV2 and PCV2 genotypes with 25 strains and 30 normal serum samples, respectively, obtained from pigs. ATCC, American type culture collection, PCV2, Porcine circovirus type2.
