# Supplementary material for: Comparison of Multiplex Real-Time PCR and PCR-Reverse Blot Hybridization Assays for the Direct and Rapid Detection of Porcine Circovirus Type 2 Genotypes
Source: Front Vet Sci. 2020 Apr 30;7:200. doi: 10.3389/fvets.2020.00200 (PMC7204941; doi:10.3389/fvets.2020.00200)
Supplement: Supplementary Figure 1 — Detection limits of the multiplex real-time PCR and PCR-REBA methods evaluated using 10-fold serial diluted samples. Serially diluted PCV2-P, PCV2a/e, PCV2b, and PCV2d DNA samples ranging from 1 ng to 1 fg per reaction were used to determine the detection limit of the multiplex real-time PCR and PCR-REBA methods. In the multiplex real-time PCR assay, the amplification curve of the specific probe (A) for detecting PCV2 (R2 = 0.997), PCV2a/e probe (B), PCV2b probe (C), and PCV2d probe (D), for detecting PCV2 genotypes (R2 = 0.999) are shown. The overall detection limit of this assay for the PCV2 genotypes ranged from ~100 to 10 fg DNA per reaction. CT was plotted against the input of the quantity of PCV2, 2a/e, 2b, 2c, 2d, and 2e DNA (repeated 40 times). The intensity of fluorescence is shown on the Y-axis (R2 = reporter signal/passive reference signal). RFU, relative fluorescence unit and R2, fluorescence units. Serially diluted PCV2-P (E), PCV2a (F), PCV2b (G), PCV2c (H), PCV2d (I), PCV2e (J), and 5 mixed co-infection PCV2 subtypes (K) with DNA amounts from 1 ng (lane 1), 100 pg (lane 2), 10 pg (lane 3), 1 pg (lane 4), 100 fg (lane 5), 10 fg (lane 6), and 1 fg (lane 7) were used to determine the detection limit of the PCR-REBA (E–J). N, negative control. PCV2c and PCV2e used synthesized DNA as a control. [file Data_Sheet_1.pdf]

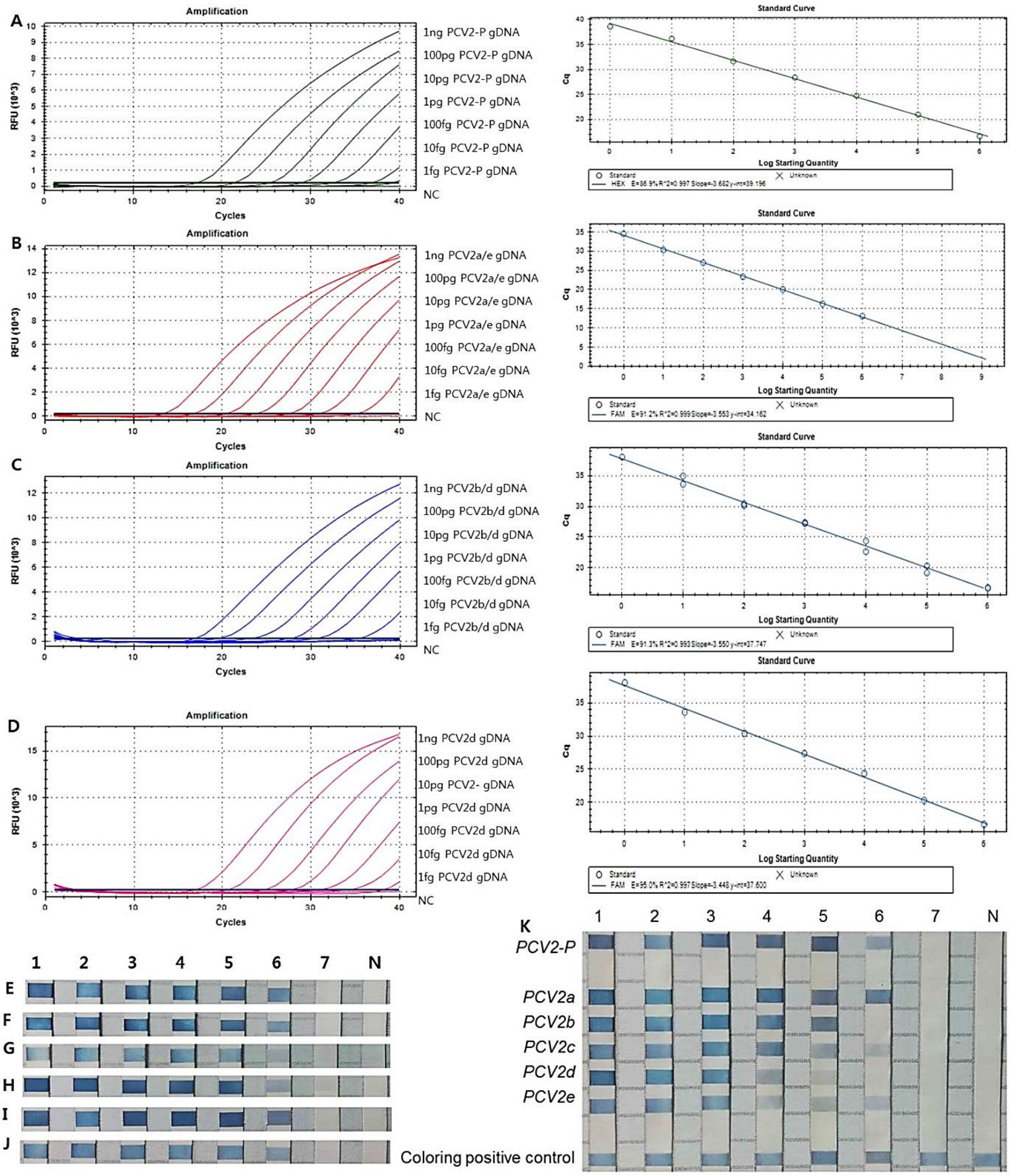

Supplementary FIGURE 1

Supplementary TABLE 1 Analytical specificity of the multiplex real-time PCR and PCR-REBA assay for detecting PCV2 and PCV2 genotypes with 25 strains and 30 normal serum samples, respectively, obtained from pigs.

No.	Species	Isolate	Sample type	Multiplex real-time PCR		PCR-REBA
				PCV2 (Ct)	IC (Ct)	
1	*Porcine reproductive and respiratory syndrome virus*	Field isolate	Tissue	N/A	24.31	N/A
2	*Porcine reproductive and respiratory syndrome virus*	Field isolate	Tissue	N/A	24.66	N/A
3	*Porcine reproductive and respiratory syndrome virus*	Field isolate	Tissue	N/A	24.33	N/A
4	*Hemophilus parasuis*	Field isolate	Tissue	N/A	22.46	N/A
5	*Hemophilus parasuis*	Field isolate	Tissue	N/A	24.53	N/A
6	*Salmonella typhi*	ATCC 19430	Culture	N/A	23.41	N/A
7	*Salmonella enteritidis*	ATCC 13076	Culture	N/A	24.41	N/A
8	*Mycoplasma hyopneumoniae*	Field isolate	Tissue	N/A	24.28	N/A
9	*Mycoplasma hyopneumoniae*	Field isolate	Tissue	N/A	24.21	N/A
10	*Mycoplasma hyopneumoniae*	Field isolate	Tissue	N/A	24.5	N/A
11	*Porcine rotavirus*	Field isolate	Tissue	N/A	24.37	N/A
12	*Porcine epidemic diarrhea virus*	Field isolate	Stool	N/A	24.57	N/A
13	*Porcine epidemic diarrhea virus*	Field isolate	Sludge	N/A	24.31	N/A
14	*Porcine Parvovirus*	Field isolate	Collagen	N/A	24.31	N/A
15	*Porcine circovirus type 3*	Field isolate	Tissue	N/A	24.41	N/A
16	*Porcine circovirus type 3*	Field isolate	Tissue	N/A	24.38	N/A
17	*Porcine circovirus type 3*	Field isolate	Tissue	N/A	24.53	N/A
18	*Porcine circovirus type 3*	Field isolate	Tissue	N/A	24.26	N/A
19	*Porcine circovirus type 3*	Field isolate	Tissue	N/A	24.05	N/A
20	*Porcine circovirus type 3*	Field isolate	Tissue	N/A	24.69	N/A
21	*Escherichia coli*	ATCC 25922	culture	N/A	24.43	N/A
22	*Clostridium perfringens*	ATCC 13124	Culture	N/A	24.2	N/A
23	*Toxoplasma gondii*	ATCC 50853	Culture	N/A	24.12	N/A
24	Normal	Field isolate	Serum	N/A	24.52	N/A
25	Normal	Field isolate	Serum	N/A	24.12	N/A
26	Normal	Field isolate	Serum	N/A	22.25	N/A
27	Normal	Field isolate	Serum	N/A	24.36	N/A
28	Normal	Field isolate	Serum	N/A	24.24	N/A
29	Normal	Field isolate	Serum	N/A	24.27	N/A
30	Normal	Field isolate	Serum	N/A	24.25	N/A
31	Normal	Field isolate	Serum	N/A	24.34	N/A
32	Normal	Field isolate	Serum	N/A	24.34	N/A
33	Normal	Field isolate	Serum	N/A	24.37	N/A
34	Normal	Field isolate	Serum	N/A	23.44	N/A
35	Normal	Field isolate	Serum	N/A	24.32	N/A

36	Normal	Field isolate	Serum	N/A	24.27	N/A
37	Normal	Field isolate	Serum	N/A	24.3	N/A
38	Normal	Field isolate	Serum	N/A	24.38	N/A
39	Normal	Field isolate	Serum	N/A	24.47	N/A
40	Normal	Field isolate	Serum	N/A	24.38	N/A
41	Normal	Field isolate	Serum	N/A	24.13	N/A
42	Normal	Field isolate	Serum	N/A	24.34	N/A
43	Normal	Field isolate	Serum	N/A	24.4	N/A
44	Normal	Field isolate	Serum	N/A	24.4	N/A
45	Normal	Field isolate	Serum	N/A	24.38	N/A
46	Normal	Field isolate	Serum	N/A	24.4	N/A
47	Normal	Field isolate	Serum	N/A	24.49	N/A
48	Normal	Field isolate	Serum	N/A	24.32	N/A
49	Normal	Field isolate	Serum	N/A	24.27	N/A
50	Normal	Field isolate	Serum	N/A	24.29	N/A
51	Normal	Field isolate	Serum	N/A	23.09	N/A
52	Normal	Field isolate	Serum	N/A	24.29	N/A
53	Normal	Field isolate	Serum	N/A	22.58	N/A
54	*Porcine circovirus type 2*	Field isolate	Tissue	20.47	23.9	PCV2b
55	*Porcine circovirus type 2*	Field isolate	Tissue	26.85	24.55	PCV2d
56	Mixed co-infection PCV2 subtypes	plasmid	–	25.5	23.29	PCV2a, 2b, 2d mixed
57	NC	–	–	N/A	24.12	N/A

Abbreviations: ATCC, American type culture collection, PCV2, Porcine circovirus type2.
